# Common trend: move to enucleation—Is there a case for GreenLight enucleation? Development and description of the technique

**DOI:** 10.1007/s00345-014-1339-9

**Published:** 2014-06-15

**Authors:** Fernando Gomez Sancha, Vanesa Cuadros Rivera, Georgi Georgiev, Alexander Botsevski, Julian Kotsev, Thomas Herrmann

**Affiliations:** 1 ICUA, Clínica CEMTRO, Ventisquero de la Condesa 42, 28035 Madrid, Spain; 2 Hill Clinic, Alexander Pushkin Blvd. 71, Sofia, 1618 Bulgaria; 3 Medizinsche Hochschule Hannover, Hannover, Germany

**Keywords:** GreenLight laser, Benign prostatic enlargement, Enucleation, Vaporization

## Abstract

**Background:**

Transurethral laser prostatectomy has evolved as a viable alternative for the management of benign prostate enlargement. Since the renaissance of laser prostatectomy with the advent of the holmium:yttrium–aluminum–garnet laser in the 1990s, various lasers and subsequent procedures have been introduced. These techniques can be categorized as vaporizing, resecting, and enucleating approaches. Photoselective vaporization of the prostate (PVP) is dominated by high-power lithium triborate (LBO) crystal lasers (GreenLight XPS). The mainstay of this technique is for the treatment of small to medium prostate volumes whereas enucleating techniques, such as holmium laser enucleation of the prostate and thulium enucleation of the prostate, focus on large-volume glands. In order to perspectively “delimit” LBO into the field of large-volume prostates, we developed LBO en bloc enucleation to render it as a competing transurethral enucleating approach.

**Materials and methods:**

We present a detailed stepwise progressive technique developed in Madrid, Spain, for the complete removal of the transitional zone by vapoenucleation. The steps include exposition of the prostatic capsule by PVP toward the peripheral zone, thereby identifying the anatomical limits of enucleation. Subsequently, the transitional zone is excised in a single bloc and morcellated after its placement into the bladder.

**Conclusion:**

This new GreenLight en bloc enucleation technique allows to treat larger prostates than those previously treated with the PVP technique.

## Introduction

Since the renaissance of lasers in the treatment of benign prostatic obstruction (BPO) secondary to benign prostate enlargement (BPE) after the neodymium: yttrium–aluminum–garnet (Nd:YAG) era, laser prostatectomy has been employed for almost two decades with beneficial results. A range of laser has been employed involving different wavelengths, power capacities, and modes of action. The current European Association of Urology (EAU) guidelines on non-neurogenic male lower urinary tract symptoms (LUTS) recommend both holmium:YAG (Ho:YAG) laser enucleation (HoLEP) and photoselective vaporization of the prostate (PVP; GreenLight) as minimally invasive alternatives to transurethral resection of the prostate (TURP) in men with LUTS secondary to BPO [1]. The American Association of Urology guidelines state that, furthermore, all holmium-based techniques [such as HoLEP, holmium laser resection of the prostate (HoLRP), and holmium laser ablation of the prostate (HoLAP)] as well as PVP are appropriate and effective treatment alternatives to TURP [2]. This is supported by the EAU guidelines on lasers and technologies also evaluating thulium-based resection/enucleation techniques [thulium:yttrium–aluminum–garnet (Tm:YAG) laser prostate vaporesection (ThuVARP), thulium:YAG laser vapoenucleation (ThuVEP), and thulium laser enucleation (ThuLEP)] [3].

GreenLight-based PVP evolved from potassium-titanyl phosphate (KTP) 532 nm laser to 120 W LBO (GreenLight HPS) and the current 180 W LBO XPS laser system involving the MoXy Liquid Cooled side-firing fiber. PVP ablates tissue by vaporization of tissue from the prostatic urethra toward the prostatic capsule (inside-out), whereas laser energy in enucleating techniques such as HoLEP or ThuVEP [4], ThuLEP [5], and diode laser-based enucleations (eraser enucleation [6]/diode laser enucleation [7] ) is used to enter the plane of the prostatic capsule and dissect and detach the prostatic lobes (outside-in).

A meta-analysis published in 2012 revealed that equivalent clinical outcome was achieved with the GreenLight laser and TURP but with reduced likelihood of blood transfusion and clot retention with the laser treatment [8]. Surgical time was longer with the laser treatment than TURP. The most recent report comes from the GOLIATH Study in which 281 men with LUTS due BPO were randomized to treatment with the GreenLight laser with the 180 W GL XPS laser or TURP [9]. Results from the study showed that the two treatments were comparable in terms of improvement in symptoms, maximum flow rate, postvoid residual urine (PVR), and complications. Hospital stay and duration of catheterization were shorter with the GreenLight laser than TURP, and early reintervention (up to 30 days) was three times higher in the TURP group. These studies involved men with prostates sized <80 ml. One randomized controlled trial evaluated prostates sized >80 ml treated with open prostatectomy or laser therapy [10]. Results showed comparable safety and efficacy outcome between the two treatments.

## Development of an enucleation technique with the GreenLight laser

**Fig. 1:**
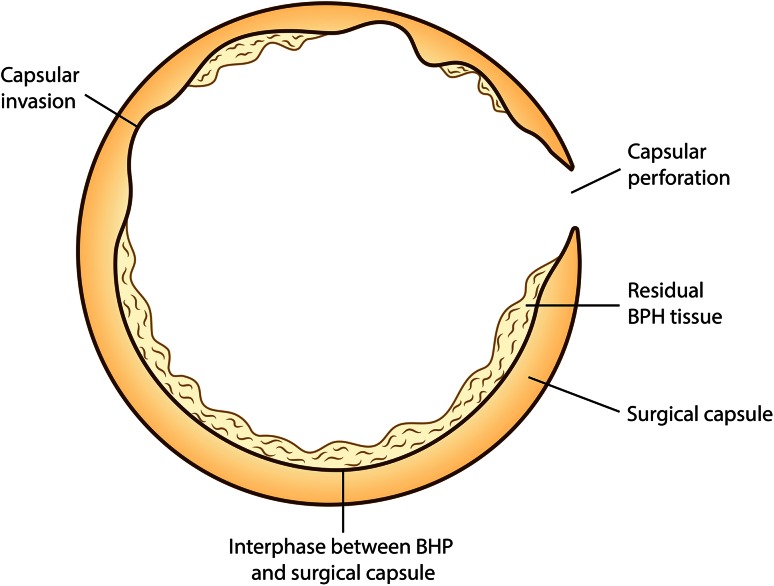
Capsule perforation or invasion caused by overaggressive vaporization. Insufficient vaporization can leave prostate tissue in place

The inherent problem of extra-anatomical approaches, such as resection or vaporization of the adenoma, is the difficulty in determining the anatomical cleavage limits, i.e., where to stop the vaporization and to understand when the prostate capsule has been reached. Attempts to vaporize the adenoma aggressively can lead to capsular violation or perforation and residual adenomatous tissue can remain, which might require retreatment or promote late secondary bleeding (Fig. 1). One of the limitations of PVP is the challenge encountered in large-volume prostates (>90 ml) in completely vaporizing the transitional zone. Hueber et al. [11] report that although prostates sized >100 ml can be treated with PVP, operating times are longer and retreatment rates can be as high as 9 %. GreenLight laser enucleation has been developed by Dr Fernando Gomez Sancha in Spain and Bulgaria to overcome these problems and has involved logical steps from standard vaporization to anatomic vaporization, then to vapoenucleation, and finally to en bloc enucleation.

## Equipment

### Laser equipment

**Fig. 2:**
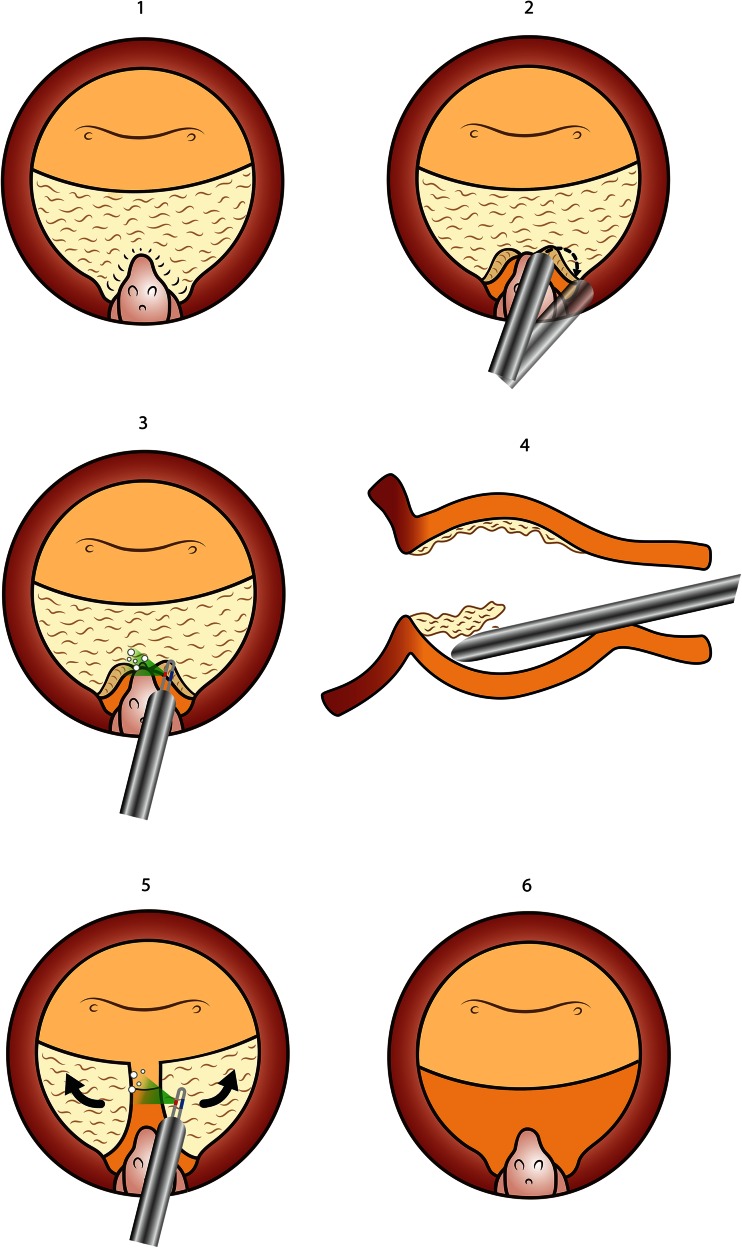
Anatomic photoselective vaporization (PVP). The starting point of this technique is a standard PVP central cavity (**1**). The capsular localization maneuver is performed in both sides using the tip of the scope and joined in the midline (**2**, **3**). Careful mechanical dissection is carried out toward the bladder neck at 6 o’clock, and a full incision of the adenomatous tissue down to capsule is completed (**4**, **5**). Vaporization is carried out laterally and anteriorly following the capsular plane (**6**)

**Fig. 3:**
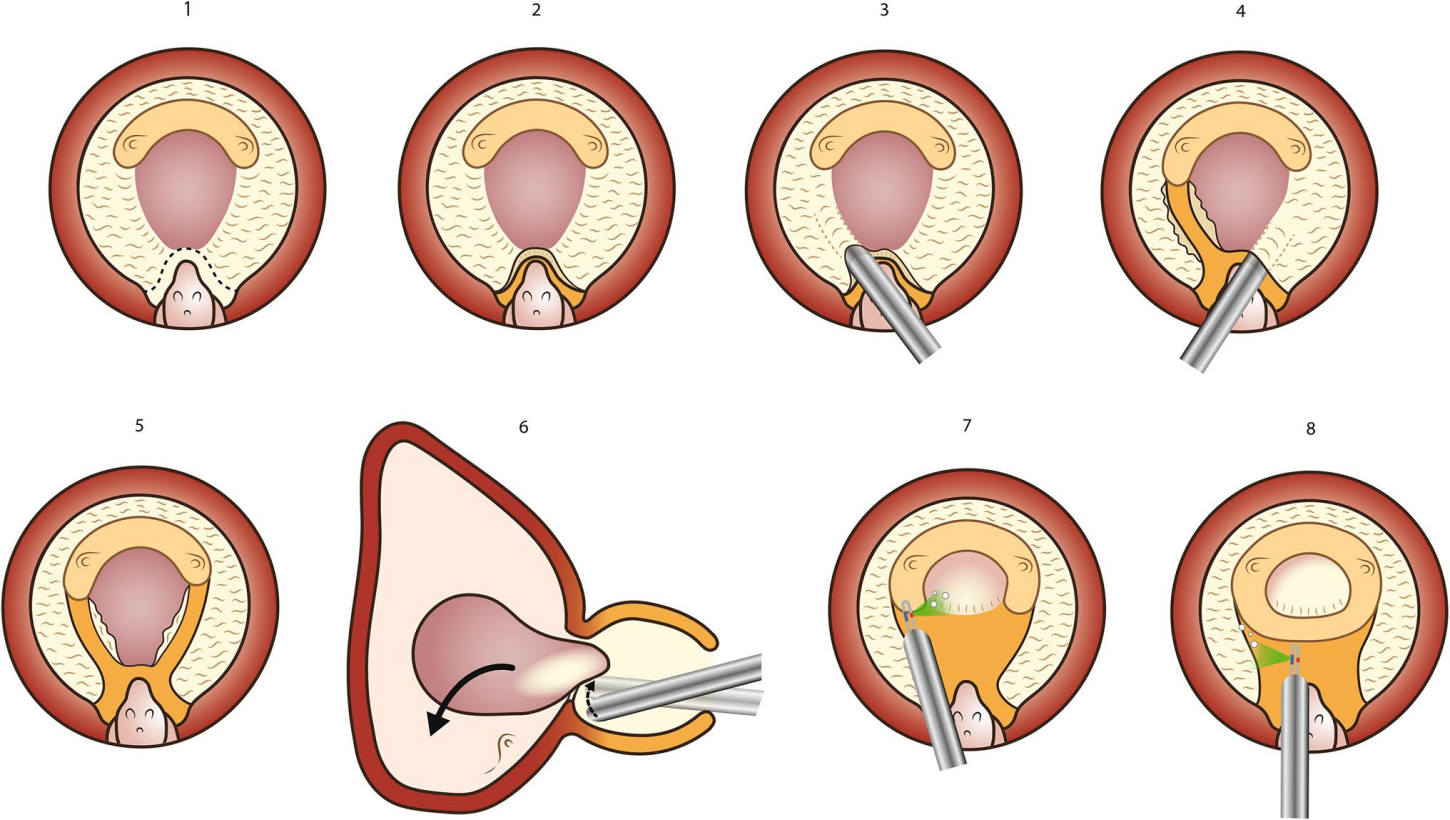
Photoselective vapoenucleation. A standard vaporization cavity is performed leaving the middle lobe intact (**1**–**3**). The capsule is localized lateral to veru montanum on both sides, and mechanical dissection of the plane is carried out toward the bladder neck at 5 and 7 o’clock (**4**, **5**). The middle lobe is dissected with the tip of the scope, and the attachments at 6 o`clock are cut to release it into bladder for subsequent morcellation (**6**). Vaporization of remaining lateral and anterior tissue is carried out following the capsular plane (**7**, **8**)

**Fig. 4:**
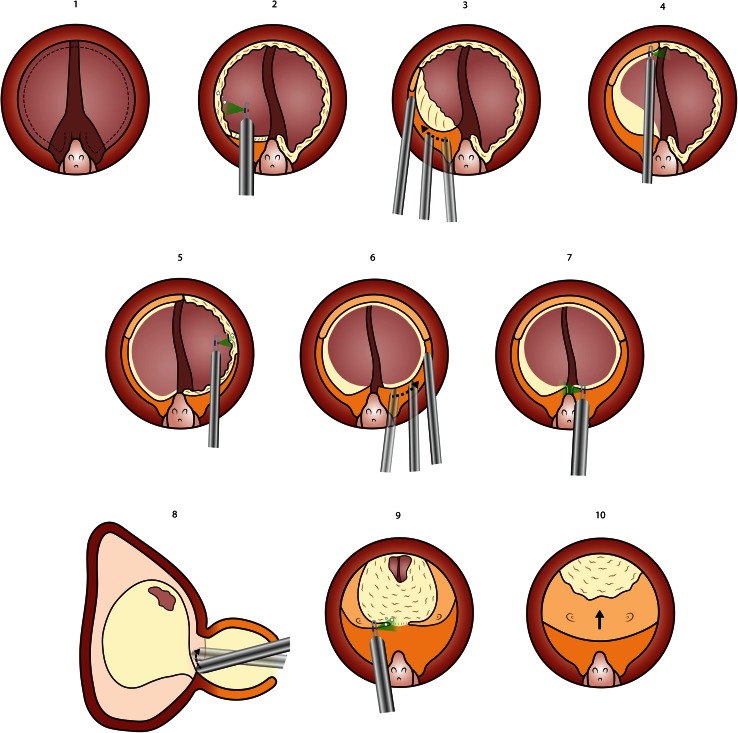
Photoselective en bloc enucleation. The procedure starts at the apex marking the “*white line*” demarcating the limit of the external sphincter (**1**). The sphincter is progressively dissected off the apex of the prostate from 6 toward 12 o’clock on one side (**3**, **4**). Mechanical dissection of the plane between capsule and lateral lobe is performed, alternating with hemostasis (**5**–**7**). The bladder is entered anteriorly and cut toward 7 and 12 o’clock (**8**). A similar process is carried out on the contralateral side, and then, the crista urethralis is cut and the posterior aspect developed with mechanical dissection until the adenoma can be flipped into the bladder (**9**). The remaining attachment at 6 o’clock is cut to release it into the bladder and morcellation follows (**10**)

**Fig. 5:**
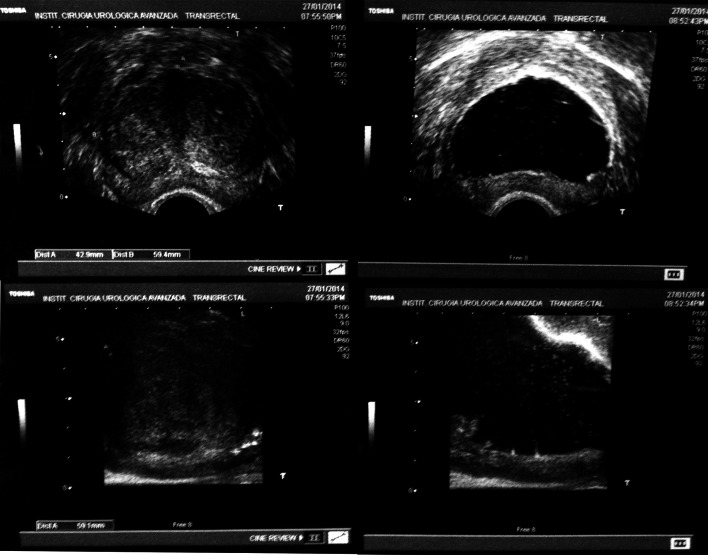
Pre- and postoperative transrectal ultrasound of the prostate showing the excellent removal of the adenoma

A 532-nm lithium triborate laser (GreenLight HPS 120 W and GreenLight XPS 180 W surgical lasers, American Medical Systems, Minnetonka, US) was used. Two different fibers were employed: the 2090 fiber and the MoXy fiber. The en bloc enucleation technique was preferably carried out with the 2090 fiber at power settings of 80 W for cutting and 40 W for coagulation. The MoXy fiber was used at a power setting of 180 W power for vaporization and of 40 W power for coagulation. The 2090 fiber was better for enucleation as the procedure needs lower power, and it was more resilient and resistant to contact vaporization, which was sometimes necessary during the enucleation technique. Also, the back of the fiber could be used to coagulate. Both anatomic vaporization and vapoenucleation require a big deal of vaporizing, and the MoXy fiber at 180 W power provided fast vaporization, and it also allowed for partial enucleation, when the power was reduced to 120 W. However, this fiber was less resistant to mechanical trauma and manipulation and could experience breakages when used for enucleation (Figs. 2, 3, 4, 5).

### Endoscopy equipment

A 26-F continuous flow resectoscope (Richard Wolf, Germany) with a special laser bridge for the laser fiber was used; a 30° down lens was preferred. It is important to use a resectoscope with a traumatic flat end or a smooth edge in order to avoid capsular perforation when the mechanical dissection was carried out with the shaft of the resectoscope. A straight laparoscopic camera (Richard Wolf, Germany) was connected to the telescope for image enlargement and recording of procedures. Saline solution was used for irrigation throughout the procedure, keeping the height of the bags at 40–60 cm except for the morcellation phase (80–100 cm for distension of the bladder at physiological intravesical pressure).

### Morcellator

A mechanical tissue morcellator (Piranha: Richard Wolf GMbH, Knittlingen, Germany) was used for intravesical morcellation of fragments. The new disposable Vmax single use rotation blades (Richard Wolf GMbH, Germany) were used in conjunction with the motor, control unit, foot pedal, and suction pump. A morcelloscope/nephroscope was adapted to the external sheath of the resectoscope in order to allow introduction of the morcellator blade into the 26-F continuous flow resectoscope (Richard Wolf GMbH, Germany). A double inflow and higher saline bag height was preferable to keep the bladder distended so that bladder wall injuries were avoided during morcellation.

## Surgical technique

Three stages of surgical technique are described, which represent the stepwise learning process.

### Patient preparation

The patient is placed in the lithotomy position as for a TURP. After sterilization of the skin and surgical draping, sterile anesthetic gel is instilled into the urethra. Dilatation or Otis urethrotomy is performed if needed. Under direct vision, a 26-F continuous flow resectoscope is inserted in the urethra avoiding urethral or prostatic trauma and bleeding that can make the procedure more difficult to perform. A straight laparoscopic camera is preferred in order to avoid interference with the laser fiber. The outflow must always be opened to avoid bladder overdistension. Keeping the irrigation fluid bags low lowers the pressure in the operative field and promotes better intraoperative hemostasis and it also lowers the risk of fluid absorption. To keep the bladder distended, the bags must be elevated for the morcellation phase. A careful cystoscopy is carried out to rule out bladder problems and to visualize the ureteral orifices. The anatomy of the sphincter and prostatic urethra is visualized as it is important for surgical planning.

### Stage 1: Anatomic photoselective vaporization

To improve standard photoselective vaporization of the prostate (PVP), the depth of vaporization is based on the anatomical localization of the capsule. This “anatomical PVP” performs a central cavity in the prostate with the standard technique for vaporization as described previously [12]. The next step is to localize the capsule at the apex of the adenoma by carrying out a bilateral incision lateral to the veru montanum and then using the tip of the shaft/beak of the resectoscope to push laterally and find and develop the interphase between capsule and adenoma. This capsular localization maneuver is paramount for the later development of GreenLight laser en bloc enucleation. Hemostasis of bleeding vessels is carried out during the procedure. As both lateral planes are exposed, these planes are connected medially proximal to the veru montanum. Careful mechanical dissection of the plane with the resectoscope is followed toward the bladder neck at 6 o’clock, alternating with low-power coagulation of any capsular bleeding. A 6 o’clock incision is made by firing the laser upwards from below the adenoma to vaporize adenomatous tissue. This allows visualization of the capsule from the veru montanum to the bladder neck and will serve as the anatomic reference of the capsule for the remainder of the procedure. Vaporization is then carried out laterally in both sides and anteriorly with the knowledge of the precise depth of the capsule. Intraoperative transrectal ultrasound is conducted to check that all the adenomatous tissue has been removed.

### Stage 2: Photoselective vapoenucleation

The vapoenucleation hybrid technique allows the surgeon to familiarize himself with the capsular anatomy and with the mechanical dissection maneuvers that need to be carried out for the development of the anatomical plane between adenoma and capsule. A standard vaporization technique is carried out leaving the median lobe intact. Five and 7 o’clock incisions are carried out without attempting to reach the surgical capsule. The capsular localization maneuver is performed and instead of dissecting the plane at 6 o’clock, the dissection follows the direction of the previous incisions, toward 5 and 7 o’clock. The remaining adenomatous tissue is vaporized to create two grooves at 5 and 7 o’clock that reach the surgical capsule. Then, the median lobe is dissected mechanically and flipped into the bladder. If the median lobe is left attached at 6 o’clock, it can be resected with bipolar or monopolar TURP bloodlessly, or if it is released into the bladder, it can be morcellated. This small-volume morcellation is usually carried out in outstanding visibility conditions and serves well as a learning curve for morcellation.

### Stage 3: Photoselective en bloc enucleation

A circumferential incision is carried out to mark the limit between the apex of the adenoma and the external sphincter, respecting the veru montanum. The *crista urethralis* is not cut. This “white line” will serve as a landmark for sphincter preservation throughout the procedure. The capsular localization maneuver is carried out on the right side, and mechanical enucleation is carried out using the tip of the scope to develop the virtual space between surgical capsule and adenoma, alternating gentle dissection maneuvers with coagulation at low power of any capsular bleeding. Of note, the recognition of the capsular plane is easier than with holmium laser as the capsule is not affected by mechanical pulse energy, and thus, the anatomy is more recognizable. Progressively, the apex is carefully liberated from the sphincter by cutting at 80 W power. The dissection is aimed ventrally and the bladder is spontaneously entered at 11 o’clock just by following the anatomical plane between capsule and adenoma. Then, the bladder neck is incised toward 12 and 7 o`clock. Once the right side is released completely from the capsule, the same steps are carried out on the other side. When all lateral and anterior aspects of the adenoma are free, the cresta urethralis is cut at 6 o’clock and the posterior aspect is liberated. It then becomes possible to flip the adenoma into the bladder by lifting it and pushing it carefully with the scope. The 6 o’clock attachment at the bladder neck is cut to deliver the adenoma “en bloc” into the bladder. Morcellation is carried out to finish the procedure. The urinary catheter can be removed the morning after all these surgical variants.

## Discussion

The possibility of using the GreenLight laser in an enucleating approach was first discussed in 2010. The procedure consisted of an initial vaporization of the anterior zone, particularly the para-sphincteric areas at 11, 12, and 1 o’clock in order to simplify the subsequent enucleative procedure [13]. The technique of GreenLight laser photoselective vapoenucleation described in the current manuscript involves a gradual learning path. Surgeons can start by just localizing the capsule for anatomic vaporization, then moving on to perform partial enucleations, and then, when they have developed the necessary skills and have sound anatomical knowledge, they can perform the whole en bloc enucleation procedure. Also interestingly, they are trained to perform morcellation of tissue with small pieces at first, with very good visualization conditions and then move on to more complex cases. One of the potential advantages of using a GreenLight enucleation procedure is that it allows the surgeon to switch between resection and vaporization at any time during the procedure if needed during the learning curve.

Brunken et al. [14] reported a feasibility study on GreenLight laser enucleation involving 21 men with mean (SD) prostate size 75 (38) ml. The technique employed enucleating the median lobe of the prostate using vaporization of deep channels at the 5 and 7 o’clock positions from the bladder neck to the veru montanum down to the surgical capsule and then connecting the channels. The excised tissue was dissected toward the bladder neck. Vaporization of tissue lateral to the veru montanum down to the capsule followed. The lateral lobes were then dissected and tissue removed with a morcellator. The mean (SD) operative time was 112 (27) min, and intraoperatively, there was one capsule perforation. Catheterization time was 1.2 (0.4) days and hospitalization was 3.6 (0.9) days. The procedure allowed the removal of 35 (220) g prostate tissue, which was equivalent to 47 % of the total prostate weight. Improvements in symptom score and PVR were reported at the mean (SD) follow-up of 5.8 (1.8) months.

Since 1998, transurethral enucleation is performed using a Ho:YAG laser, which operates at 2,140 nm in a pulsed manner and can be used to enucleate (HoLEP) or resect (HoLRP) the prostate. The wavelength employed by the laser is strongly absorbed by water, and for this reason, the area of tissue coagulation that results is limited to 3–4 mm, which reduces blood loss [15]. In terms of efficacy, a number of studies have been published providing long-term outcome data up to a mean of 6.1 years revealing durable outcome [16]. A randomized controlled trial comparing transurethral resection of the prostate with HoLRP has indicated comparable symptomatic and urodynamic improvements, but a significantly longer mean resection time with the laser treatment (42.1 vs. 25.8 min) [17]. Results from two meta-analyses show comparable symptomatic improvement with TURP and HoLEP but longer operating time with laser therapy [18, 19]. In terms of safety of this laser, no critical complications have been reported. Peri-operative dysuria rates of around 10 % have been published [5, 15, 20]. Post-operative complications include retrograde ejaculation (75–80 %) [15]. Early postoperative urinary incontinence rates reported range from 8.3 % to 44 % [21–23]. The meta-analyses revealed a significantly shorter catheterization time and hospital stay with HoLEP than TURP as well as reduced blood loss and fewer blood transfusions, but a longer operation time [18, 19]. One of the perceived limitation to the use of the holmium laser is the need for specialist endoscopic skills and a long learning curve.

The thulium:yttrium–aluminum–garnet (Tm:YAG) laser can be used in four different modes: vaporization (ThuVAP), vaporesection (ThuVARP), vapoenucleation (ThuVEP), or enucleation (ThuLEP). Mattioli et al. [24] reported good outcome when the laser operating at 70 W power in a vaporization technique was used to treat 99 patients with prostates sized <35 ml. Symptomatic improvement out to 9 months and reduced PVR were reported. The majority of the data on the Tm:YAG laser focus on its use in a resecting mode. In common with the Ho:YAG laser, the Tm:YAG laser has a shallow penetration and excellent hemostasis. Operating in a 2 µm continuous wave mode causes increased vaporization capacity, resulting in resection combined with tissue vaporization [25, 26]. Vaporesection with the Tm:YAG laser has been compared in a randomized controlled trial with TURP and shown to produce comparable clinical outcome but with reduced morbidity, in particular, reduced bleeding and shorter hospitalization and catheterizations times [26]. ThuVEP has been compared with the HoLEP and shown to have comparable short-term results with reduced blood loss with the ThuVEP technique [4]. Gross et al. [5] have also reviewed outcome in 1,080 patients treated with ThuVEP at a single institution and found the procedure to be effective and safe. A more recent modification of the technique involves ThuLEP, in which the laser is used to incise the prostate tissue to the level of the capsule and enucleate prostate lobes that are later morcellated [27].

Enucleation with the GreenLight laser has some differences to HoLEP and ThuLEP. GreenLight uses a 70° angle side-firing fiber, while the holmium and thulium lasers employ an end-firing fiber. HoLEP and ThuLEP enucleate the adenoma in two or three pieces in contrast to GreenLight laser en bloc enucleation, which enucleates the prostate in one piece. The main advantage of mechanical enucleation with the GreenLight laser is that the anatomy is not altered at all by the effect of energy on tissue. Coagulation of the capsule at 40 W is performed after the plane has been dissected, and this helps in the recognition of the right plane for enucleation. Also, the amount of energy the surgical capsule receives is relatively low, and this might result in a reduced likelihood of postoperative dysuria. Another potential advantage is that gentle mechanical dissection keeps the surgical plane in the interphase, reducing the chance of capsular invasion or perforation and minimizing the occurrence of sinus opening and saline absorption.

## Conclusion

The GreenLight laser has demonstrated excellent vaporization and coagulation properties in prostatic tissue. The standard vaporization technique has some limitations in terms of gland size that can be treated. The techniques described in this paper allow the surgeon to learn how to perform complete removal of larger prostate glands in a stepwise fashion, allowing for a progressive learning curve until he/she can perform en bloc enucleation and morcellation.

## Abbreviations

EAU: European Association of Urology
PVP: Photoselective vaporization of the prostate
TRUS: Transrectal ultrasound of the prostate
HoLAP: Holmium laser ablation of the prostate
HoLRP: Holmium laser resection of the prostate
Ho:YAG: Holmium:yttrium–aluminum–garnet
Tm:YAG: Thulium:yttrium–aluminum–garnet
Tm:VARP: Thulium laser prostate vaporesection
ThuVEP: Thulium:YAG laser vapoenucleation
ThuLEP: Thulium laser enucleation
